# Supplementary material for: Sex-Dependent Effects of Glutamatergic Disruption on Dopaminergic Neuron Subtype Vulnerable in Parkinson’s Disease
Source: bioRxiv. 2026 May 11:2026.05.06.723291. Preprint. [Version 1] doi: 10.64898/2026.05.06.723291 (PMC13192589; doi:10.64898/2026.05.06.723291)
Supplement: Supplement 1 [file media-1.pdf]

Title: Sex-Dependent Effects of Glutamatergic Disruption on Dopaminergic Neuron Subtype Vulnerable in Parkinson's Disease

Authors: Kathleen F. Carmichael[1, 2], Victor M. Martinez Smith[1], Jinhui Ding[3], Gavin Riccobono[1], Lisa Chang[1], Lixin Sun[1], Lupeng Wang[1], and Huaibin Cai[1*]

Supplementary Figures and Tables

Supplementary Fig. 1

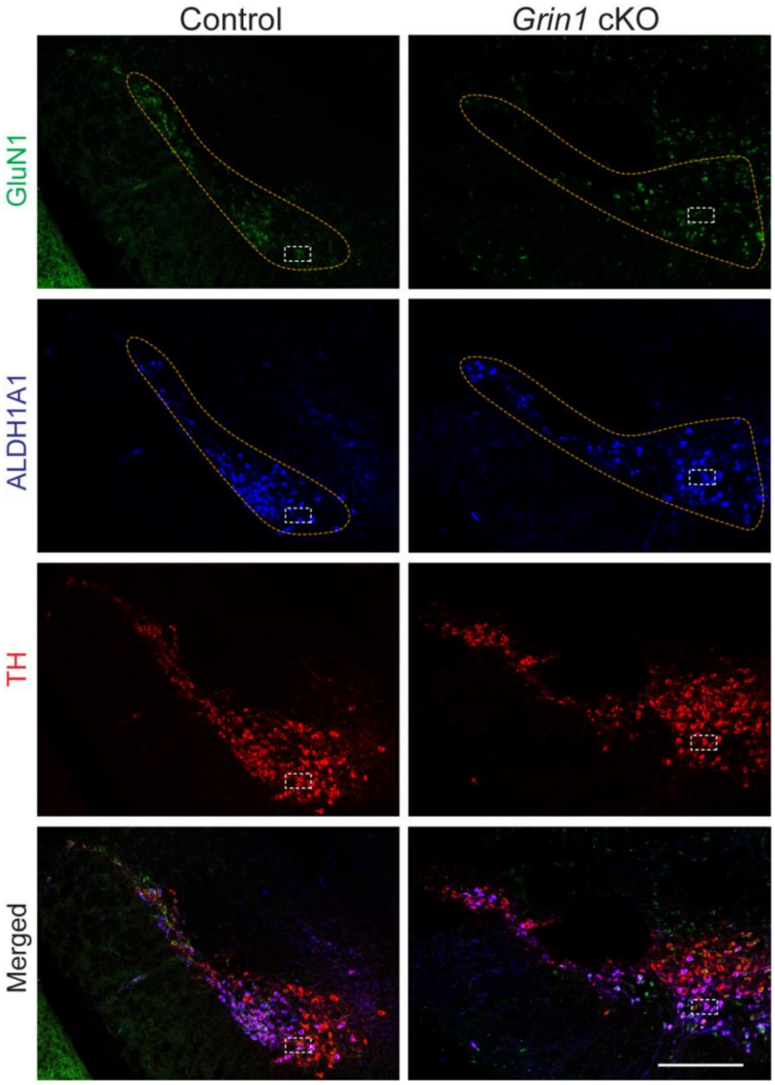

Supplementary Fig. 1 Genetic deletion of *Grin1* in ALDH1A1$^+$ DANs.

Representative image (20×, single z-plane) showing GluN1 (green), ALDH1A1 (blue), and TH (red) co-staining in *Grin1* cKO and control midbrain DANs outlined by orange dotted lines. White doted boxes indicate regions shown in Fig. 1b. Sale bar, 500 μm.

Supplementary Fig. 2

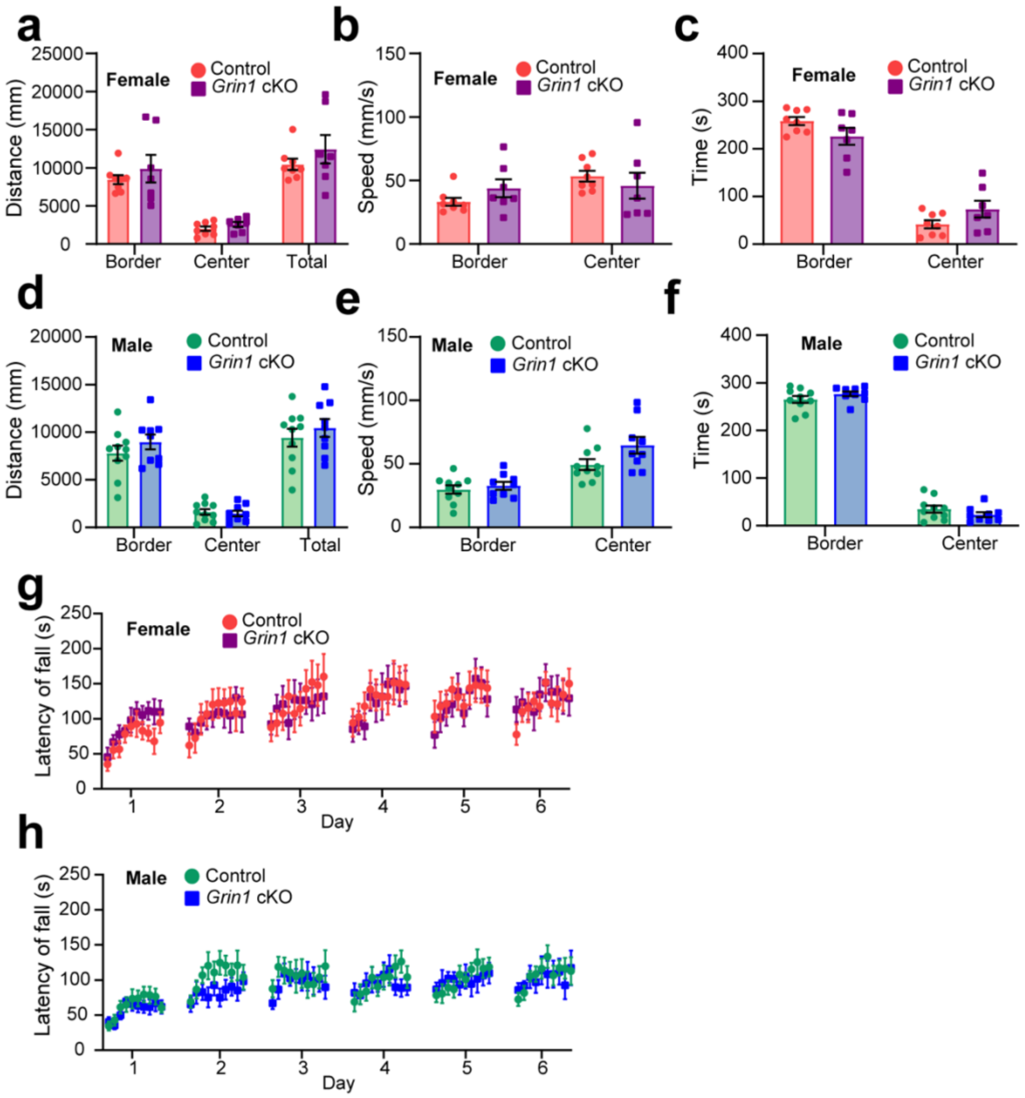

Supplementary Fig. 2 No sex difference in spontaneous locomotion or motor learning.

a, Distance traveled (mm) in the border, center, and total regions of the arena by female Grin1 cKO (n = 7) and control mice (n = 8). Unpaired t-tests: border, p = 0.4722; center, p = 0.2295; total, p = 0.3476.

b, Average speed (mm/s) in the border and center regions by female *Grin1* cKO (n = 7) and control mice (n = 8). Unpaired t-tests with Welch's correction: border, p = 0.2063; center, p = 0.5126.

c, Time spent (s) in the border and center of the open field arena by 3–4-month-old female *Grin1* cKO (n = 7) and control mice (n = 8) during a 5-min session. Unpaired t-tests with Welch's correction: border, p = 0.1382; center, p = 0.1385.

d, Distance traveled (mm) in border, center, and total regions by male *Grin1* cKO (n = 9) and control mice (n = 10). Unpaired t-tests with Welch's correction: border, p = 0.3085; center, p = 0.7143; total, p = 0.4490.

e, Average speed (mm/s) in border and center regions by male *Grin1* cKO (n = 9) and control mice (n = 10). Unpaired t-tests with Welch's correction: border, p = 0.5286; center, p = 0.0718.

f, Time spent (s) in border and center regions by 3–4-month-old male *Grin1* cKO (n = 9) and control mice (n = 10). Unpaired t-tests with Welch's correction: border, p = 0.2072; center, p = 0.2027.

j, Rotarod performance across 6 days (10 trials/day) in 3–4-month-old *Grin1* cKO (n = 7 females, 10 males) and control mice (n = 8 females, 12 males). Two-way ANOVA, genotype: F(1,35) = 0.7043, p = 0.7043.

k, Rotarod performance in female *Grin1* cKO (n = 7) and control mice (n = 8). Two-way ANOVA, genotype: F(1,13) = 0.0001432, p = 0.9906.

l, Rotarod performance in male *Grin1* cKO (n = 10) and control mice (n = 12). Two-way ANOVA, genotype: F(1,20) = 0.4647, p = 0.5033.

All data are presented as mean ± SEM.

Supplementary Fig. 3

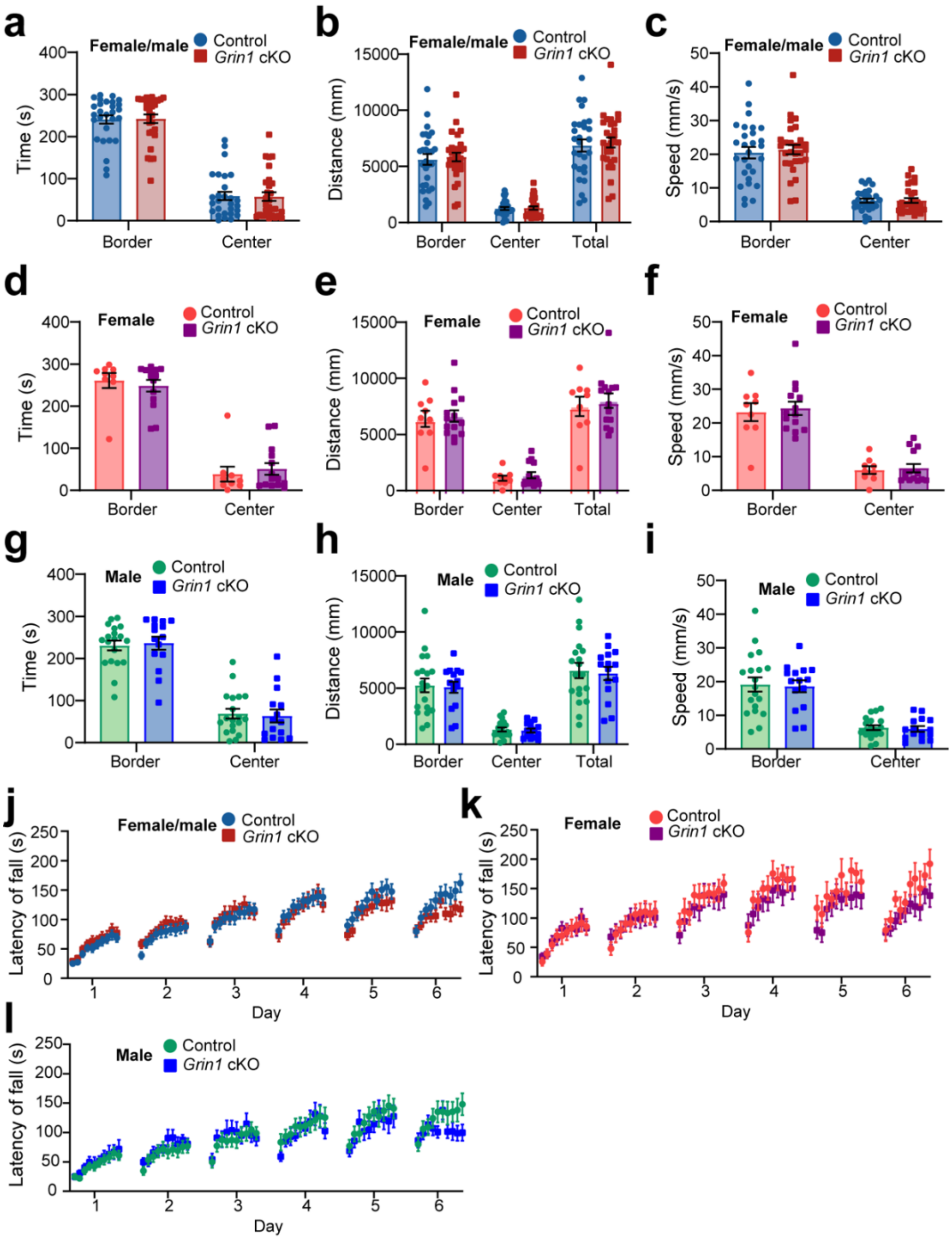

Supplementary Fig. 3 Aged (15+ months) *Grin1* cKO mice display no deficits in spontaneous locomotion or motor learning.

a, Time spent (s) in border and center regions of the open field arena by 15+ month-old (range 15–27 months) *Grin1* cKO (n = 14F, 15M) and control mice (n = 9F, 19M) during a 5-min session. Unpaired t-tests with Welch's correction: border, p = 0.9061; center, p = 0.9066.

b, Distance traveled (mm) in border, center, and total regions. Unpaired t-tests with Welch's correction: border, $p = 0.7165$; center, $p = 0.8289$; total, $p = 0.7032$.

c, Average speed (mm/s) in border and center regions. Unpaired t-tests with Welch's correction: border, $p = 0.6788$; center, $p = 0.9750$.

d, Time spent in border and center regions by aged female *Grin1* cKO ($n = 14$) and control ($n = 9$) mice. Unpaired t-tests with Welch's correction: border, $p = 0.5873$; center, $p = 0.5873$.

e, Distance traveled (mm) in border, center, and total regions by aged females. Unpaired t-tests with Welch's correction: border, $p = 0.7827$; center, $p = 0.4689$; total, $p = 0.6400$.

f, Average speed (mm/s) in border and center regions by aged females. Unpaired t-tests with Welch's correction: border, $p = 0.7330$; center, $p = 0.7368$.

g, Time spent in border and center regions by aged male *Grin1* cKO ($n = 15$) and control ($n = 19$) mice. Unpaired t-tests with Welch's correction: border, $p = 0.7783$; center, $p = 0.7788$.

h, Distance traveled (mm) in border, center, and total regions by aged males. Unpaired t-tests with Welch's correction: border, $p = 0.8395$; center, $p = 0.7077$; total, $p = 0.7808$.

i, Average velocity (mm/s) in border and center regions by aged males. Unpaired t-tests with Welch's correction: border, $p = 0.8404$; center, $p = 0.7234$.

j, Rotarod performance across 6 days (10 trials/day) in 15+ month-old *Grin1* cKO (n = 13F, 15M) and control mice (n = 9F, 20M). Two-way ANOVA, genotype: $p = 0.8056$.

k, Rotarod performance in aged females. Two-way ANOVA, genotype: $p = 0.5185$.

l, Rotarod performance in aged males. Two-way ANOVA, genotype: $p = 0.8588$.

All data are presented as mean ± SEM.

Supplementary Fig. 4

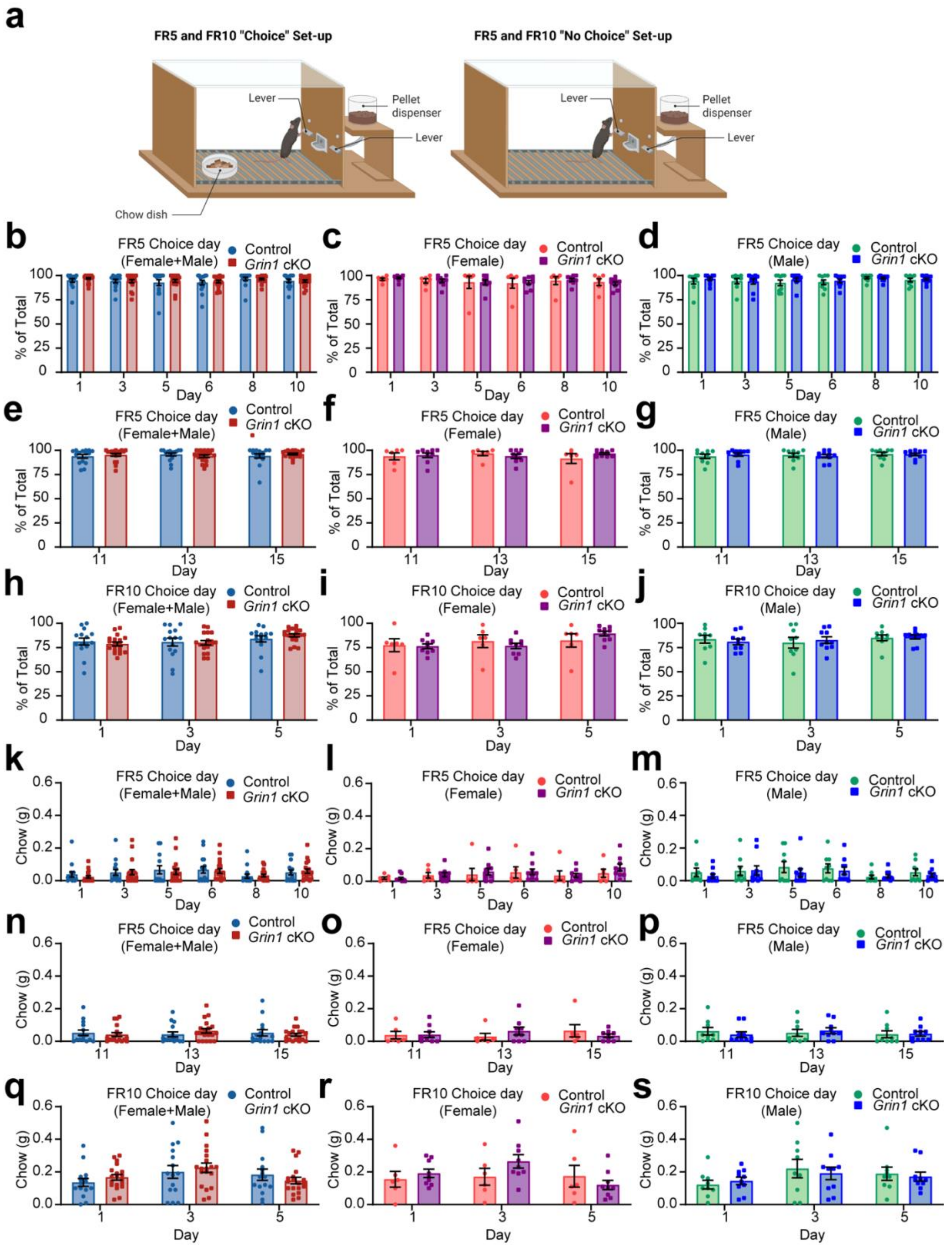

Supplementary Fig. 4 Mice overwhelmingly choose to work for pellet rewards rather than consume freely available chow under high-effort choice conditions.

a, Schematic of operant chamber setup for Choice and No Choice sessions, created by BioRender.

b–d, Percentage of total food consumed derived from chocolate pellet rewards on each FR5 Choice Day with a 50-pellet limit by Grin1 cKO (n = 9F, 10M) and control mice (n = 6F, 9M) for all mice (b), females only (c), and males only (d).

e–g, Percentage of total food consumed derived from chocolate pellet rewards on each FR5 Choice Day without a pellet limit for all mice (e), females only (f), and males only (g).

h–j, Percentage of total food consumed derived from chocolate pellet rewards on each FR10 Choice Day without a pellet limit for all mice (h), females only (i), and males only (j).

k–m, Chow consumed on each FR5 Choice Day with a 50-pellet limit for all mice (k), females only (l), and males only (m).

n–p, Chow consumed on each FR5 Choice Day without a pellet limit for all mice (n), females only (o), and males only (p).

q–s, Chow consumed on each FR10 Choice Day without a pellet limit for all mice (q), females only (r), and males only (s).

All data are presented as mean ± SEM.

Supplementary Fig. 5

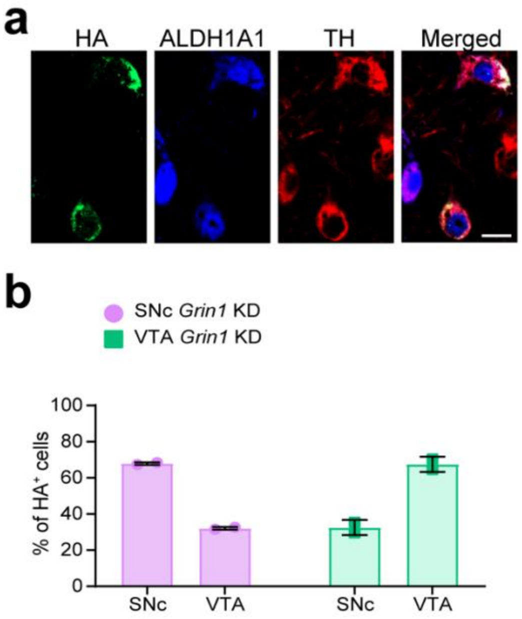

Supplementary Fig. 5 Partial but regionally preferential knockdown of *Grin1* in SNc and VTA ALDH1A1⁺ DANs.

a, Representative confocal images (63×, single z-plane) showing HA (green), ALDH1A1 (blue), and TH (red) co-staining in midbrain coronal sections from *Aldh1a1*$^{+/P2A\text{-}CreERT2}$ mice injected with AAV9-FLEX-SaCas9-U6-sg*Grin1*. Scale bar, 20 µm.

b, Quantification of the percentage of HA^+ neurons among $ALDH1A1^+$ neurons in the SNc and VTA of *Grin1* KD mice. Data are presented as mean ± SEM.

Supplementary Fig. 6

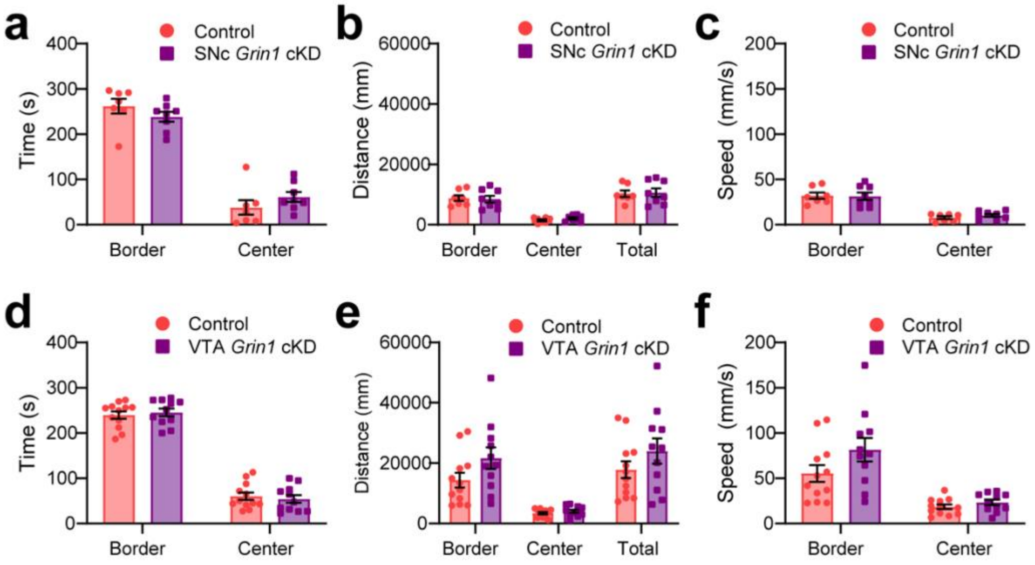

Supplementary Fig. 6 Neither SNc nor VTA *Grin1* knockdown impairs spontaneous locomotion.

a–c, Time spent (s) in border and center regions (a), distance traveled (mm) in border, center, and total regions (b), and average velocity (mm/s) (c) in female SNc *Grin1* KD mice (n = 8) and female controls (n = 7) during a 5-min open field session.

d–f, Time spent (s) in border and center regions (d), distance traveled (mm) in border, center, and total regions (e), and average velocity (mm/s) (f) in female VTA *Grin1* KD mice (n = 11) and female controls (n = 12) during a 5-min open field session.

All data are presented as mean ± SEM. Statistical comparisons were performed using unpaired t-tests. No significant changes were found.

Supplementary Table 1 Female *Grin1* cKO vs. Control Post-Restriction (padj < 0.05, Fold Change (FC) > 1.2)

GeneName	*baseMean*	*log2FC*	*padj*
H2-K1	274.048027	0.49638252	0.00640695
Col11a2	316.441569	0.45390244	0.03854266
Qdpr	2566.85077	0.45273172	4.68E-09
Mag	2146.60956	0.4473795	0.00640695
Col16a1	355.722652	0.43007071	0.03854266
Apoe	12023.6831	0.42703456	0.00012537
Cd63	905.118749	0.42293211	0.00221938
Igfbp2	305.407641	0.41292176	0.03579081
Baiap3	681.781968	0.40105077	0.01573579
Phgdh	622.164414	0.40072661	0.00221938
Gfap	2621.36072	0.3962146	0.04380223
Olig1	1103.20849	0.39371649	0.01512989
R3hcc1	280.236116	0.39143261	0.03060983
Pltp	503.965327	0.38862777	0.04249842
Itm2a	714.325178	0.38302008	0.01677498
Lpar1	471.072691	0.37922501	0.04333716
Guk1	1397.98688	0.37805874	0.04497013
Slc7a10	372.2885	0.36270342	0.02222805
Cmtm5	428.322447	0.35771038	0.04497013
Bloc1s1	526.122997	0.35492153	0.04333716
Cnp	3266.37051	0.34814951	0.00059299
Ndufb7	771.499959	0.34764064	0.03945405
H2-D1	616.357482	0.33041766	0.04380223
Phldb1	1365.46801	0.32373267	0.00640695
Mid1ip1	1092.51039	0.31629337	0.01524657
Pink1	5885.473	0.3075992	0.00059299
Bcan	2329.94115	0.30278552	0.01868968
Hnrnpa0	1269.29602	0.29554033	0.03060983
Ache	1062.43659	0.29458411	0.04333716
Cadm4	2330.2018	0.29012105	0.02222805
Sec11c	1018.76529	0.28584088	0.02300283
Bsg	3888.13154	0.27461338	0.00960625
Pum2	1867.35034	-0.2671986	0.03854266
Cnot7	998.164703	-0.2870596	0.04380223
Appl1	858.349384	-0.2892618	0.01856791
Kcnj3	952.222791	-0.289777	0.04984066

Ppm1e	1646.11248	-0.2979408	0.04333716
Mtpn	3092.61337	-0.2988075	0.04333716
Rock2	1378.07972	-0.3131995	0.02237311
Far1	775.517807	-0.314367	0.04096246
Pten	1670.37988	-0.3236781	0.01652385
Ralgps2	410.00751	-0.3281221	0.04380223
Spred1	1793.12301	-0.3350164	0.00525725
Nrn1	1414.88207	-0.3370176	0.01856791
Fut9	970.81522	-0.353677	0.04333716
Bmi1	562.704336	-0.3559021	0.04096246
Hook1	655.269096	-0.3595119	0.01512989
Tut4	374.173951	-0.3642117	0.03854266
Lrrtm2	573.647381	-0.369953	0.01041116
Eeig2	560.46551	-0.3722001	0.02802366
Kdm7a	561.548439	-0.3798213	0.01652385
Nrip3	1658.79198	-0.3808398	0.01652385
Zfp654	314.373061	-0.3878257	0.04333716
Hycc2	915.786244	-0.3930666	0.01652385
Mal2	1228.56137	-0.394907	0.01380553
Ankrd45	546.709824	-0.3954765	0.01780307
Fgd4	266.679158	-0.396174	0.04497013
Tiam2	563.895236	-0.4018757	0.01677498
Nlk	731.232006	-0.4019342	0.00347561
Lrrc7	365.567793	-0.4119464	0.01791048
Fam81a	1549.34139	-0.4395466	0.00834767
Homer1	967.815243	-0.458026	0.00083814
Ipcef1	608.751061	-0.4773606	0.00655203
Clk1	1761.61205	-0.5358466	0.01780307
Rnpc3	471.854253	-0.5379202	0.00221938
Aldh1a1	1716.97002	-0.753976	6.67E-06

Supplementary Table 2 Female vs. Male *Grin1* cKO Post-Restriction (padj < 0.05, FC > 1.2)

GeneName	***baseMean***	***log2FC***	***padj***
Opalin	199.874916	0.94074985	0.04999425
Mgp	161.580948	0.67253799	0.00039338
Cd59a	195.462801	0.61694186	0.00910119
Ppp1r14a	166.63343	0.60682339	0.00222927
Pdlim2	215.289431	0.55880949	0.00235
Pllp	504.51742	0.53079076	1.63E-05
Trf	3705.59642	0.51056199	2.62E-05
Fxyd1	189.142794	0.50412828	0.00692008
Qdpr	2390.4544	0.49835782	7.11E-10
Atp5me	1215.18914	0.49784423	0.00235
Snrnp25	196.175103	0.49224201	0.02784748
Uqcrh	2986.51328	0.48640747	0.00037395
Myo1d	163.166629	0.48124801	0.03320189
H1f2	247.668402	0.47866988	0.01202225
H2-K1	260.357738	0.47486901	0.00692008
Il33	644.619802	0.47231634	0.00222927
Mag	2012.45079	0.47015367	1.63E-05
Btbd17	161.53698	0.46365408	0.03581675
Gfap	2429.34713	0.45722079	0.02431769
Rps27	1885.49263	0.45612076	0.00079897
Tst	197.334377	0.4559967	0.0241288
Gstm7	200.506747	0.45370227	0.01533656
Cd63	846.298234	0.4511919	0.00011099
Mrpl2	275.828686	0.44476232	0.00609196
Rnaset2a	181.428401	0.44050004	0.02481636
Cd82	173.762047	0.42804489	0.04307917
Ndufb1	1010.6948	0.42603324	0.02061901
Anapc13	259.406356	0.42059922	0.03097916
Hebp1	249.814397	0.4175827	0.01667114
Phpt1	424.388478	0.41664707	0.02747186
Igfbp2	287.911073	0.41589941	0.01508212
C1qa	279.229178	0.41228435	0.008371
Ndufs5	926.533408	0.40866581	0.01358681
Penk	1564.37637	0.40123947	0.03911619
Cmtm5	398.878839	0.39606106	0.00707393
Syndig1l	465.000263	0.39255037	0.04205698
Ndufb7	717.729499	0.39158802	0.00141153

H2bc4	509.928065	0.39134133	0.02270128
Uqcr11	806.573092	0.38555179	0.00235
Bloc1s1	491.264356	0.38434903	0.00270135
Bola2	358.501684	0.38258403	0.02508383
Alg5	210.548904	0.38162031	0.04023602
Rpl36	1199.50634	0.38086685	0.03023662
Phlda3	263.437569	0.38007597	0.04585676
Bckdha	362.343364	0.37790632	0.03735992
Rpl38	2180.00411	0.37432007	0.00444585
Apoe	11527.3077	0.37267928	0.00141153
Atp5mf	1594.5351	0.37171686	0.03959799
Tmem256	601.267093	0.37150623	0.04310578
Cox6c	3831.05859	0.36866871	0.00222927
Rps29	2356.49297	0.36757092	0.00235
Mog	782.921298	0.36450358	0.00235
Guk1	1323.71794	0.36390309	0.03288297
Nme1	897.508809	0.36272577	0.00281137
Ctss	444.886186	0.36250566	0.01724156
Ddit4	1038.32883	0.35868748	0.02533006
Phgdh	595.266276	0.35631965	0.00235
Rps9	3090.92583	0.35586348	0.00316792
Cst3	8616.19169	0.35505611	0.00039338
Nudt18	316.984724	0.35238449	0.03248714
Naa38	318.677929	0.35185594	0.04347668
Mt3	2221.37983	0.35079473	0.00270135
Arsg	355.362921	0.34877974	0.03345851
mt-Nd4l	1742.82083	0.34760821	0.01190589
Cox7a2	2343.44043	0.34692908	0.02489946
Cnpy2	377.138635	0.34647336	0.02224116
S100a1	615.003761	0.34563186	0.01939294
Etfb	295.111922	0.345497	0.02784748
Ddrgk1	510.801159	0.34426685	0.00726593
Ndufa5	786.929057	0.34406408	0.00351926
Rps17	2332.01649	0.34380526	0.00107041
Ndufa2	653.057473	0.34278164	0.00476122
Slc35b2	376.533957	0.34269586	0.03693368
Rpl31	2286.49486	0.34167959	0.0057383
Rpl11	3273.06556	0.33983803	0.00848102
Plekhb1	7262.1324	0.33471872	0.04311491
Ndufa13	895.83493	0.33460388	0.02481636

Rpl37	1926.6503	0.33123862	0.04536576
Cldn10	344.244722	0.3309476	0.04999425
Naxd	419.628352	0.32935794	0.03959799
Olig1	1062.15109	0.32720673	0.03345851
Ramp1	832.150714	0.32644702	0.03133398
Rida	415.849514	0.32643231	0.04205698
S100b	2983.31911	0.32383379	0.04023602
Cldn11	1549.8037	0.32315673	0.00762478
Tmem63a	386.001098	0.32040423	0.04114355
Npc2	811.705375	0.31755809	0.02277991
Bsg	3618.39569	0.31721824	0.00039338
Ndufa3	1010.46075	0.31439631	0.01491784
Rpl36al	555.159784	0.31038991	0.03133398
Ndufb9	2909.26404	0.31033819	0.02067908
Sec11c	954.515685	0.31002765	0.04921266
Fau	2772.26924	0.30925653	0.04063552
Nnat	1657.96569	0.30776773	0.00075996
Slc7a10	356.959247	0.3064793	0.04205698
Gpr37	682.606424	0.30622763	0.01091217
Polr2e	767.347193	0.30469489	0.02127614
Tubb2b	799.66616	0.3008951	0.00955448
Rps8	3970.91495	0.29818337	0.00242693
Rps28	1109.56787	0.29683404	0.04336012
Cox5a	1086.22385	0.29497217	0.0124189
Cnp	3134.58024	0.29435216	0.00051371
Ptn	2236.2406	0.29408069	0.00716801
Mrps33	766.54243	0.29170123	0.03735992
Hcn2	2172.60828	0.28968612	0.03459921
Uqcrq	1591.38384	0.28817619	0.03133398
Rasgrp2	568.888709	0.28802514	0.03459885
Clta	1640.09319	0.28617846	0.01192743
Mesd	542.762661	0.28588984	0.03133398
Tmem50a	986.570912	0.28439376	0.01190589
Gpr37l1	2268.0289	0.28403024	0.00647163
Prmt1	692.261807	0.2818083	0.02747186
Mrps7	502.806085	0.27886086	0.04023602
Capns1	886.428581	0.27457747	0.0241288
Car2	1792.1873	0.27438941	0.00278746
Cd81	2595.37801	0.27016593	0.0124189
Aldh2	944.141597	0.26981238	0.03735992

Rpl37a	2226.16943	0.2676973	0.04473761
Arl6ip4	487.480269	0.26642822	0.04999425
Psmb4	1377.15057	0.26642703	0.04023602
Fabp5	996.42283	0.26638222	0.04660039
Snf8	578.652235	0.26323149	0.03832747
Spcs2	787.227968	0.26141906	0.03345851
Rpl19	4501.14433	0.26128353	0.02429898
Nktr	1447.89973	-0.2605061	0.00703059
Spred1	1643.92332	-0.2624098	0.03832747
Plxna2	987.325326	-0.2638824	0.03074449
Dusp11	826.223855	-0.2639099	0.03965821
Zfp207	1233.62465	-0.2653552	0.0241288
Nvl	716.962874	-0.2657045	0.03680651
Chl1	925.408465	-0.2666516	0.04454387
Ylpm1	877.332431	-0.2715057	0.04742388
Fryl	888.522594	-0.2715708	0.02429898
Usp34	1293.22979	-0.2716007	0.03911619
Ppm1e	1538.46219	-0.2735011	0.02330335
Nrsn1	5406.05617	-0.2752976	0.03028327
Lyst	749.05937	-0.2761712	0.02508383
Gria2	3798.83189	-0.2781645	0.00910119
Nfat5	853.577623	-0.2822747	0.03959799
Nrip3	1506.97101	-0.284056	0.03735992
Rock2	1286.69354	-0.2871329	0.04473761
Ankhd1	628.884585	-0.287384	0.04584398
Vps37a	664.661711	-0.2901274	0.04702466
Kmt2a	1808.46748	-0.2910644	0.02434852
Dennd5b	666.886824	-0.2936928	0.04311491
Tnrc6b	871.033382	-0.2956406	0.02840349
Etv1	1313.89176	-0.2959571	0.04585676
Med12l	451.797515	-0.2976669	0.03097916
Mkln1	830.841138	-0.2995261	0.02481636
Cpeb4	1004.12027	-0.2996537	0.04023602
Mga	670.533905	-0.2997963	0.03684423
Rora	937.072956	-0.2998707	0.01909128
Mapk8	847.300897	-0.3042103	0.03959799
Zfc3h1	494.069482	-0.3042639	0.04702466
Tlcd4	495.712597	-0.3046329	0.03959799
Nup153	484.770096	-0.3059496	0.02543527
Hook3	687.876442	-0.3082905	0.04999425

Shprh	472.057525	-0.3089192	0.03798956
Arfgef3	1155.92715	-0.3101574	0.02316161
Ankrd45	498.129284	-0.3104534	0.04311491
Tnr	1136.06405	-0.3125643	0.00703059
Pgm2l1	1928.85845	-0.3131839	0.02508383
Ipcef1	537.175608	-0.3141042	0.03097916
Cacnb4	1841.38136	-0.3157404	0.01419834
Dmxl1	514.551682	-0.3166876	0.02508383
Hipk3	999.120306	-0.3174596	0.00968437
Cep170	679.129646	-0.317933	0.03074449
Rabgap1l	901.391667	-0.3192435	0.01514674
Tank	328.396257	-0.3193817	0.04765109
Cpsf6	847.281986	-0.3224825	0.02067908
Braf	1041.80927	-0.3233377	0.02508383
Slf2	798.513102	-0.324079	0.00430383
Nlk	668.452987	-0.3242851	0.02326657
Far1	734.712724	-0.324944	0.02316161
Wsb1	1381.79811	-0.3252097	0.04805834
Ubr3	1731.155	-0.3271569	0.00463407
Tnpo1	644.998921	-0.3272968	0.02747186
Ankrd12	639.106482	-0.3290678	0.01202225
Rictor	642.979138	-0.3322807	0.01688371
Kmt2c	1034.08669	-0.3351662	0.00968437
Vps13a	385.939452	-0.3365287	0.04063552
Pik3c2a	334.979461	-0.3368655	0.04311491
Fut9	910.497928	-0.3384678	0.04111873
Sfpq	1252.16807	-0.3453776	0.0114219
Hook1	615.099329	-0.3454017	0.01194294
Prkaa2	335.683314	-0.3471655	0.03063038
Lrrtm2	538.335185	-0.3529534	0.03345851
Nipbl	477.845127	-0.3537171	0.01709692
Nrip1	326.917246	-0.3557632	0.02234918
Fam81a	1412.77688	-0.3568133	0.02747186
Zfp292	309.498182	-0.3571794	0.03133398
Atp11b	1229.19529	-0.357187	0.02747186
Dcp1a	236.645561	-0.3625635	0.04454387
Rfx3	330.423167	-0.3639236	0.02747186
Ppargc1a	458.645812	-0.3656208	0.01724156
Fam135b	514.962848	-0.3779701	0.02434852
Acvr2a	408.123628	-0.3822578	0.03067977

Faxc	371.918185	-0.3849505	0.01383436
Homer2	468.505728	-0.3868798	0.01839618
Hipk2	638.732817	-0.3882349	0.03735992
Tia1	1296.18244	-0.3971213	0.00235
Exph5	252.689421	-0.4129747	0.02481636
Gng4	394.572471	-0.4162777	0.02449833
Smim43	286.805525	-0.4208916	0.04753153
Phip	468.698432	-0.4221888	0.00848102
Clk1	1590.18256	-0.4271787	0.00968437
Klf12	172.484175	-0.4298338	0.04205698
Lin7a	277.659033	-0.4313439	0.02854909
Tut4	362.868084	-0.4327049	0.00235
Fgd4	257.539898	-0.4537412	0.00903222
Ndst3	351.639683	-0.4674083	0.00256019
Lcorl	186.762233	-0.4714637	0.04023602
Rnpc3	437.030373	-0.4931622	0.00235
Dlx1	184.713189	-0.49838	0.04999425
Hectd2	158.600225	-0.5469694	0.00707393
Stxbp5l	317.683406	-0.5915485	0.00059286

Supplementary Table 3 Female vs. Male *Grin1* cKO Refeeding (padj < 0.05, FC > 1.2)

GeneName	*baseMean*	*log2FC*	*padj*
Crtam	159.218579	0.89397572	0.00022375
Ppp1r17	253.988641	0.72578874	0.00200338
Car8	2498.4762	0.72240612	0.02547201
Pvalb	1846.61872	0.65055657	2.15E-07
Cd59a	171.043912	0.63824539	0.00234615
Rpa3	154.101342	0.60316832	0.01127369
Sar1b	813.837586	0.59885807	0.00083282
Gm56450	422.422412	0.54903475	0.00268575
Nrep	1843.68119	0.54179683	0.00087596
Selenof	2470.11471	0.53837754	0.00022375
Sumo1	1798.66746	0.51711939	0.00088785
Ciao2a	443.934127	0.51378241	0.00705974
Smim26	296.23253	0.5129004	0.01483541
Mkks	204.729255	0.50741723	0.00359634
Ggh	225.183061	0.50693699	0.01367739
Cox16	225.809947	0.50303026	0.02331741
Tm2d1	543.3849	0.5026828	0.00922603
Cep15	329.349667	0.49816659	0.00248676
Smim8	174.196141	0.49782562	0.03479579
Ube2v2	1412.36935	0.49709547	0.04375039
Pigx	181.337623	0.49620722	0.03563207
Acyp1	338.912758	0.49077026	0.0092212
Haus1	123.351236	0.48798978	0.01844832
Ccdc90b	260.427153	0.48791754	0.00524867
Gng11	221.121133	0.48706103	0.01181282
Ndufs4	1317.49169	0.48599827	0.00087596
Medag	259.081283	0.48538178	0.00093949
Gng13	459.923368	0.48324629	0.00456836
1110059E24Rik	379.561152	0.47178511	0.01844832
A430005L14Rik	229.731683	0.47166086	0.0226924
Zfp560	122.160199	0.46959317	0.03584175
Stk17b	125.017982	0.46771851	0.02990791
Rpp30	148.576706	0.46750802	0.01483541
Cript	764.976106	0.46585894	0.0033047
Hypk	171.056194	0.46238356	0.03414936
Lyz2	208.628607	0.4623632	0.03562166
1810037I17Rik	819.777108	0.45948579	0.00308567

Smim15	671.155631	0.45876694	0.00248676
Mien1	610.301249	0.45777964	0.00142688
C330018D20Rik	189.314933	0.45718468	0.02415697
Cox7c	2204.17876	0.45675723	0.00200338
Slirp	405.851958	0.45632079	0.0212338
Psma5	1152.31907	0.45422354	0.00532254
Tmem126a	255.366978	0.45405048	0.04230427
C1d	736.283195	0.45086345	0.01558959
Arl6	285.523504	0.45072207	0.00248676
Tma7	1327.7082	0.44907872	0.00456836
Eef1e1	287.557117	0.44831658	0.00560632
Zbtb26	129.455755	0.44768482	0.02239612
Itgb1bp1	465.920718	0.44602269	0.01090116
Gm2007	193.921486	0.44583367	0.02529826
Ndufaf4	400.84964	0.44485018	0.00292376
Etfrf1	366.302934	0.44456897	0.0054127
Mrpl14	258.52657	0.44374077	0.01782609
Nup35	179.643367	0.44059955	0.02565907
Mrpl20	731.915987	0.43900275	0.00429778
Ndufb6	1147.40063	0.43825918	0.00456836
Ccdc126	137.873245	0.43815097	0.01795213
Nts	142.939001	0.43592648	0.03847688
Zfp874a	182.540693	0.4352959	0.01441162
Med21	362.104708	0.4349063	0.01127369
Eif3e	935.98704	0.4345202	0.00650312
Sptssa	492.682882	0.43396571	0.02645887
Bcap29	568.697009	0.43370519	0.00256791
Cebpzos	286.796739	0.43353978	0.01777441
Gabarapl2	3885.27501	0.4326535	0.00090963
Glmn	169.870204	0.43222724	0.01092879
Tnfaip6	176.417534	0.43213772	0.01827321
Gm10033	214.413761	0.43192727	0.01368721
Timp4	822.324868	0.43113181	0.00222097
Lamtor5	686.511083	0.43064319	0.00892933
Lsm3	204.582162	0.43016525	0.01514005
Ift20	806.802467	0.42696983	0.00452523
Pde5a	176.457537	0.42575551	0.03666908
Acyp2	373.127797	0.42464294	0.02415697
Nop10	584.236908	0.42463627	0.01476885
Cops9	1380.97414	0.42360315	0.0082968

Amn1	176.988828	0.42273631	0.02897421
Ostf1	208.690729	0.41871075	0.01090116
Med6	229.704434	0.41823943	0.01777441
Rpl24	2066.71519	0.41757455	0.00136166
Cetn3	733.928284	0.41755636	0.00705974
Cstb	289.097502	0.41701453	0.02298864
Ndufa7	1224.30585	0.41668021	0.00952846
Clybl	242.093748	0.41581342	0.01302628
Sdhaf4	297.866665	0.41537224	0.03703514
Prdx1	1920.88334	0.41428575	0.00827754
Cnih1	938.586799	0.41351181	0.00313367
Ap3s1	576.247138	0.41345369	0.00369641
Neurod1	600.583283	0.41306103	0.03032892
Elof1	647.76233	0.41047047	0.01661174
Tmem60	244.226434	0.40872778	0.01181282
Commd6	470.862519	0.40819977	0.02483492
Mtln	343.088648	0.40792099	0.01068415
Mrps21	379.412961	0.406916	0.0092212
Rnf138	168.909236	0.4067512	0.02395583
Mrps14	361.788696	0.40557257	0.00705974
Tomm5	515.828915	0.40514023	0.00200338
Ostc	430.031465	0.4048656	0.0251625
Vamp8	124.351381	0.40274332	0.04542842
Mrpl33	406.279793	0.40130045	0.03502705
Vps29	1934.91502	0.40122281	0.00222097
Dph3	555.546808	0.40120073	0.00862459
Alkbh7	201.127463	0.40118599	0.04629228
Mrpl34	154.59011	0.4007841	0.04296245
Mrpl13	473.240494	0.40036263	0.01022591
Psmc6	1158.66011	0.40003435	0.00827754
Commd1	282.932957	0.39905922	0.00862459
Fcf1	233.966688	0.39625636	0.0146222
Eif4a2	11679.7303	0.39536538	0.00202836
H3f3a	2507.32916	0.39400986	0.01441162
Sec61g	673.219848	0.39372494	0.03562166
Spryd7	668.792657	0.39131381	0.0046996
Dusp19	238.75865	0.39093097	0.03465087
Hopx	820.126682	0.39005186	0.02072567
Rpl35a	3012.92519	0.38922381	0.01483541
Gng10	581.453629	0.38871657	0.01368721

Ndufc2	1313.36515	0.38530469	0.00569148
Eif2s2	1085.85455	0.3836793	0.00452523
Mreg	166.61177	0.38311541	0.04542842
Mob4	723.321762	0.38265528	0.0263376
Bnip3	1712.04543	0.38198338	0.00359634
Dbi	2005.46447	0.38155932	0.0161822
Bbip1	592.210771	0.38092237	0.04625252
Sav1	170.436827	0.38087356	0.03230583
Cryzl1	889.07824	0.38002843	0.00387091
Bnip3l	2220.90116	0.37868501	0.00093949
Chpt1	914.081217	0.37818535	0.00234615
Sdhaf3	218.447251	0.37668692	0.0256611
Snap23	217.337079	0.37616719	0.0294758
Septin7	4576.53842	0.37511218	0.00034827
Zfp874b	202.017854	0.37455319	0.03666908
Gm14325	242.011731	0.37432275	0.03470854
Naa20	697.621987	0.37419101	0.00862459
Cops3	665.420873	0.37384234	0.00369641
Hspe1	936.64685	0.37304637	0.02790403
Dctn3	980.63447	0.3715825	0.00433875
Crls1	366.812584	0.37093937	0.02790403
Ost4	406.461724	0.3704957	0.01110346
Polb	453.205696	0.36999242	0.00237524
Mrpl41	765.946499	0.36852729	0.03841817
Naca	2830.21904	0.36835479	0.01155129
Chrac1	199.999496	0.3683459	0.03045413
Tmed5	280.616309	0.36769903	0.00859357
Rtraf	831.971282	0.36767826	0.03281345
Uqcc2	1072.67192	0.36511761	0.01777441
Emc6	438.062871	0.36500508	0.00859357
Rap1a	662.166471	0.36457736	0.00271299
Coa6	213.233474	0.36323583	0.04324808
Rps20	1955.15052	0.36124318	0.0212338
Ndufc1	536.208685	0.36114638	0.04243815
Tmem242	774.198097	0.35929727	0.0054127
S100a1	589.736293	0.35823551	0.02089038
Ifrd1	433.595935	0.35714727	0.0161822
Scoc	1662.5468	0.35674584	0.00170948
Sec22a	188.847681	0.35631277	0.03179971
Erh	751.250896	0.3556153	0.01782609

Gnpda2	222.008057	0.35419774	0.03840906
Cln5	238.588931	0.35342828	0.02747102
Arl1	1961.82501	0.35200315	0.00165976
Uqcrq	1378.36687	0.35197737	0.01239244
Mrps33	727.451692	0.35129816	0.01558959
Lamtor3	532.662263	0.35093923	0.0092212
Btg1	557.221075	0.35021081	0.02498746
Fkbp3	1777.47498	0.35019076	0.00136166
Rps10	2549.91638	0.35008069	0.02581572
Commd8	592.750761	0.34975845	0.01023318
Aspa	276.407434	0.34974973	0.02236469
Ccng1	838.028612	0.34951854	0.00268553
Nck1	252.84717	0.34946137	0.02635808
Nsmce2	222.815654	0.34933072	0.04314042
Umad1	511.309007	0.34797843	0.01155129
Skp1	5446.5927	0.34733267	0.00452523
Mrpl21	258.514007	0.34634112	0.02793778
Csgalnact2	226.960003	0.34632539	0.01912429
Grpel1	579.656278	0.3460206	0.00696551
Selenok	1164.92445	0.34585698	0.0092212
Sf3b6	616.198644	0.34549927	0.04593503
Lcorl	189.884626	0.34531047	0.04683485
Mrpl53	422.367295	0.34530196	0.01558959
Bud31	488.82627	0.34502248	0.02285662
Selenot	2891.26573	0.3447606	0.00094084
Echs1	905.565763	0.34453625	0.00230684
Rpl5	4656.53789	0.34450738	0.02646808
Acer3	268.314213	0.3436206	0.03160297
Nr1d2	1941.63642	0.34294086	0.00022375
Etv1	1376.43674	0.34291572	0.00034827
Chmp2b	595.211662	0.34269297	0.0092212
Atp5if1	1607.52386	0.34261508	0.02107446
S100a16	1060.88903	0.34257009	0.0290849
Chmp2a	920.381086	0.3423677	0.00551414
Glrx3	1091.94796	0.34168105	0.00369641
Ppia	11045.0805	0.3415795	0.00862459
Tmem128	286.049508	0.34049699	0.03666908
Nae1	522.413133	0.34028312	0.00541513
Rps15a	2276.10767	0.33971866	0.02635808
Ddt	444.242207	0.33935646	0.04669783

Gpx1	1002.50858	0.33782404	0.0256611
Cfl2	738.247118	0.33703698	0.00862459
Erg28	303.519928	0.33681122	0.01558959
Mob1a	342.004155	0.33660928	0.01441162
Eif3m	955.345305	0.33581809	0.00859357
Pnrc2	681.174367	0.33538355	0.01825661
Capza2	2847.64007	0.33472458	0.00093949
D3Ertd751e	192.04424	0.33431053	0.03785501
Cycs	2070.03369	0.33340537	0.00230684
Fpgt	195.197309	0.33291325	0.04542842
Arpc5	1146.74606	0.33257301	0.01782609
Eef1akmt2	203.200849	0.33193969	0.04858859
Mrpl50	523.673714	0.33167029	0.04512373
Ube2b	1451.42624	0.33157121	0.00256791
Rap1b	762.887739	0.33081979	0.01656208
Ier3ip1	587.351109	0.32915081	0.02142217
Hint1	1557.20198	0.32886744	0.0402499
Atp5pf	1786.32452	0.32874373	0.00313367
Zfp35	214.06583	0.32869999	0.04264951
Atp5pd	2609.17086	0.32839739	0.00862459
Bloc1s2	338.068658	0.32828166	0.01782609
Blvra	212.033553	0.32791034	0.04324808
Atp6v1g1	1220.86671	0.32757083	0.00862459
Btf3l4	925.091018	0.32730025	0.00202836
Mrpl11	460.642233	0.32704916	0.03479579
Mrpl46	307.549487	0.32658675	0.04683059
Snrnp27	300.749868	0.32645669	0.04267558
Tmem256	498.404505	0.32623061	0.04882284
Selenow	6472.57077	0.32622867	0.01242449
Zfp120	194.295254	0.32576297	0.0462101
Cacybp	834.794309	0.32554765	0.00248676
Smim20	269.882238	0.32485305	0.0370572
Supt4a	540.957244	0.32441412	0.03997118
Zfp938	240.662187	0.32381487	0.03074496
Nt5c3	491.874707	0.32358818	0.03977528
Rpl27	1545.7976	0.32344789	0.03230327
Tomm7	673.397108	0.32335465	0.04127241
Tvp23b	412.131316	0.32332193	0.02453069
Aasdhppt	272.832677	0.32310958	0.02285662
Synpr	1689.64251	0.32282064	0.00200338

Golga7	1441.42759	0.32251309	0.00670618
Kitl	496.855907	0.3216419	0.00705974
Cibar1	494.193142	0.32147791	0.0261588
Idi1	732.146283	0.32114678	0.00844262
Cox14	772.644357	0.32112796	0.02415713
Lsm1	246.706845	0.32089769	0.03767933
Nudt19	885.611225	0.32001817	0.04314042
Tmem68	409.782531	0.31987154	0.02529826
Cav2	423.380059	0.31984506	0.02616402
Rpl41	3326.49389	0.31940521	0.04797401
Pomp	1149.41559	0.31929266	0.00862459
Eloc	1269.97716	0.31879703	0.02456346
Anapc11	654.266255	0.3185567	0.00705974
Rgs5	891.657986	0.3185538	0.00065432
Btf3	1668.78569	0.31595779	0.00886749
Iqcb1	352.414774	0.3157051	0.04011688
Mrps18c	366.948526	0.31467456	0.02325308
Zfp617	415.01615	0.31458064	0.03445062
Thoc7	680.13105	0.31457536	0.00952846
Dram2	258.795561	0.31427007	0.04629228
Cisd2	718.73876	0.31383353	0.00862459
Pex13	411.878576	0.31376104	0.02298864
Yipf5	580.968632	0.312361	0.03076348
Skic8	484.707667	0.31235792	0.01110346
Guk1	1224.12752	0.31090523	0.00821264
Pkia	2289.41198	0.31087981	0.00988317
Txndc9	470.786905	0.31079344	0.0092212
mt-Co2	53666.4974	0.30973943	0.01777441
Eef1b2	1821.97943	0.30948795	0.01661174
Rpl36al	511.437947	0.30941763	0.0161822
Psma1	858.197791	0.30933543	0.01321133
Vamp3	448.604726	0.30836004	0.02857625
Ndufa8	1374.00026	0.30827452	0.01827321
Smim7	1267.65285	0.30822053	0.00827754
Hikeshi	385.396236	0.30671918	0.02897071
Phospho2	340.893489	0.30637921	0.02453069
Tmem33	1136.69876	0.30617865	0.00652799
Brk1	1619.5886	0.30574017	0.03465087
Ptcd3	582.580535	0.30530746	0.02325308
Cops4	961.762793	0.30495901	0.0092212

Atp5mf	1439.3844	0.30476068	0.04324808
Lrrc57	325.087446	0.3046379	0.03666908
Atp6v1f	864.346653	0.3043134	0.0088412
Crbn	952.410439	0.3037334	0.00862459
Ndufb8	2029.52357	0.30348897	0.02453069
H2az2	428.60173	0.30312218	0.03801882
Higd1a	1019.36759	0.30310598	0.01724057
Pex7	360.379992	0.3019668	0.02529826
Uqcrb	766.804322	0.30191416	0.01441162
Clk1	1505.56741	0.30146667	0.03465087
Pno1	318.317295	0.30137607	0.03181119
Lztfl1	531.210756	0.30087439	0.01844832
Arpp19	2710.13912	0.30065191	0.00705974
Mrps23	475.157628	0.30044755	0.01476885
Sub1	2009.09466	0.30034479	0.01302628
Serp1	583.989702	0.30001552	0.02242778
mt-Atp6	33168.162	0.29970862	0.01441162
Atp5f1c	2738.47114	0.29921367	0.04924873
Polr1d	575.446373	0.29919679	0.01734963
Tomm22	691.564192	0.29863891	0.0172333
Sec11c	1028.05033	0.29820722	0.02498746
Zfand6	580.434198	0.29719959	0.02285662
Slc25a33	665.280092	0.29709331	0.02545871
Crot	384.615925	0.29707907	0.03702189
Mpc1	1413.15156	0.29616514	0.0088412
Tmem70	486.223727	0.29584944	0.02142217
Ap4s1	410.803382	0.29539541	0.03814931
Paip2	1674.79092	0.29514659	0.01476885
Ptcd2	296.42592	0.29503519	0.04264951
Cops2	987.179392	0.29495222	0.01537298
Ran	2482.29532	0.29449061	0.00222097
Rps11	2510.52289	0.29298513	0.04504846
Thap12	1281.05972	0.29225081	0.0141086
Tmed2	1348.57366	0.29220402	0.00862459
Rps14	3224.65547	0.29216643	0.0479108
Cd63	868.251573	0.2921154	0.02325308
Micos10	1011.70923	0.29190676	0.01479725
Mrpl24	466.821185	0.29140798	0.02236469
Mpc2	1119.66831	0.29138836	0.03323118
Pcna	346.04723	0.29082897	0.02079349

Cox5b	2879.06232	0.29035937	0.0212338
Phpt1	411.128396	0.28978302	0.02874252
Sft2d1	342.832705	0.28952914	0.04394977
Rpl19	4010.55159	0.28915374	0.01825661
Olfm3	482.436917	0.28793034	0.01123544
Nrsn1	5207.39379	0.28726666	0.02656964
Svip	1055.58708	0.28696702	0.03830159
Ndufb9	2421.53896	0.28685488	0.02498746
Lsm6	532.401329	0.28679013	0.01090116
Ergic2	522.067529	0.2863405	0.01656208
Rnf7	1116.15537	0.28613266	0.02415697
Mff	2779.74008	0.28550963	0.03072986
Rps5	2365.02929	0.2848731	0.03830159
Nipa2	322.957351	0.28441522	0.04629228
Ptp4a1	1468.50895	0.28434157	0.01633498
Mob1b	320.403186	0.28404609	0.03465087
Hsbp1	2352.04133	0.28327543	0.01782609
Tceal1	1086.11673	0.28325654	0.0161822
Ssu72	616.792682	0.2828016	0.01782609
Rsl24d1	603.305486	0.28178722	0.02034553
Esd	784.729661	0.28147197	0.04489412
Dcun1d5	329.896533	0.28135096	0.03294305
Dnajc15	970.958849	0.28082649	0.02812222
Timm23	881.167482	0.28056153	0.02176595
Gria4	1688.00595	0.28036535	0.00859357
Arrdc3	721.65127	0.27875927	0.0443231
Psma4	748.186432	0.27821104	0.02236939
Sdhd	1109.24515	0.27813758	0.04182945
Map1lc3b	3226.13401	0.27777582	0.01023318
Uqcrc2	2538.39378	0.27743348	0.00369641
Jkamp	539.648588	0.27735109	0.0212338
Srsf7	1139.61292	0.27661131	0.04392684
Atp5po	2352.34484	0.27635594	0.02903652
Lin7c	780.263653	0.27630513	0.00248676
Zfp68	431.951006	0.27613473	0.04011688
Fgfr1op2	1136.76737	0.27575394	0.01042543
Ift22	579.479912	0.27551159	0.01844103
Cggbp1	618.648002	0.27518123	0.01305719
Ndufab1	999.159482	0.27460178	0.02498746
Zranb2	2045.92624	0.27432176	0.00359634

Mrpl48	1075.71071	0.27353333	0.01915434
Ppp1cb	2976.9788	0.27345317	0.00067601
Cdc26	408.151285	0.27327042	0.04519672
Zc2hc1a	571.926294	0.27314883	0.01827321
Chchd2	3115.04261	0.27246947	0.04683059
Vmp1	1308.04761	0.27228456	0.00433875
Dpy19l4	372.867179	0.27197485	0.0393252
Gmfb	2044.83492	0.27081904	0.00675485
Rpl7l1	515.645891	0.27066042	0.03977182
Atp6v0b	2738.91837	0.26987421	0.02757035
Pdcd6	686.94009	0.26911611	0.04201752
Mat2b	1376.78153	0.26841356	0.01827321
Zcrb1	531.277491	0.26798303	0.02498746
Ggps1	783.517887	0.26755333	0.01825661
Zfp260	603.045151	0.2670305	0.02027538
Ufm1	746.57179	0.26680199	0.03850775
Chordc1	577.942992	0.26670559	0.01368721
Ube2d3	2807.17373	0.26620343	0.03479579
Atp5pb	3235.43373	0.26613771	0.02415697
Cox6c	3369.56719	0.26509351	0.03302955
Cct4	1210.91579	0.26480982	0.01402437
Sdhaf2	514.885218	0.26329489	0.04512373
Sumo2	1422.88986	0.26294388	0.03767933
Tsg101	626.395416	0.26273585	0.03766312
Dynll1	2055.17213	0.2626799	0.04280729
Rab11a	1141.92372	0.26226641	0.01372536
Fyttd1	943.574341	0.26212798	0.03496351
Ssr3	1604.54188	0.26084777	0.00976253
Txnl1	683.247563	0.26072678	0.01483541
Cox4i1	7742.23055	0.26070985	0.02498746
Dnajb9	439.280404	0.26030964	0.04154621
Jag2	453.05683	-0.2607194	0.02673392
Cers1	808.538518	-0.2609625	0.0212338
Ptprn	4562.69444	-0.2615375	0.00859357
Sorcs2	505.910844	-0.2631528	0.03814931
Ncor2	2087.39279	-0.263197	0.00862459
Col16a1	406.586812	-0.2638923	0.03281345
Mpp2	1172.42364	-0.2649671	0.00650312
Zmiz2	2735.76669	-0.2659478	0.01782609
Fkbp9	377.336187	-0.2671124	0.03722466

Sema6b	1236.44243	-0.2678871	0.0256611
Vat1	510.48499	-0.2695055	0.01934747
Ehmt2	1901.90448	-0.2695695	0.0092212
Nlgn2	3071.62103	-0.2704507	0.00705459
Tmem130	1959.52857	-0.2715358	0.00369641
Crtac1	493.030611	-0.2715488	0.02331741
Chrna4	502.76594	-0.2725314	0.04091326
Wfs1	1005.71785	-0.2730516	0.03332833
Tmem8b	884.172389	-0.2753159	0.02957804
Sema6c	290.909341	-0.2765339	0.04267558
Zdhhc8	973.004926	-0.277678	0.0168916
Celf5	1726.40933	-0.2778346	0.01526955
Lrfn1	474.027486	-0.2781521	0.01838863
Mbd6	604.607478	-0.2790107	0.0163617
Lrrc47	478.935867	-0.2790532	0.04293566
Lrrc4b	1686.75121	-0.2797944	0.00369641
Wipf3	1402.22731	-0.2802457	0.03582992
Scaf1	2132.58294	-0.2844543	0.00380965
Tmem63a	520.996598	-0.2847775	0.01724057
Grik5	1987.20265	-0.2849303	0.01476885
Chd3	5790.30307	-0.2850933	0.00652799
Myrf	875.878282	-0.2850974	0.00859357
Cacnb1	939.642273	-0.2851451	0.01023318
Hspa2	369.660495	-0.2855759	0.02383218
Vgf	1479.20439	-0.2875766	0.0092212
Git1	3810.11334	-0.2878027	0.0168916
Gaa	3778.84564	-0.2882305	0.00093949
Mast3	2338.61247	-0.2906477	0.01795213
Slc32a1	1491.24518	-0.2918869	0.01795213
Irf2bp1	456.414263	-0.2943563	0.04314042
Rexo1	628.993362	-0.2944944	0.00862459
Pcdh8	346.08775	-0.2949441	0.03785501
Shank3	3041.66454	-0.2960245	0.03814931
Shc2	474.822704	-0.296675	0.02176595
Zfp316	332.604468	-0.2968094	0.02812222
Lmtk3	2526.20616	-0.2970051	0.00087596
Tnk2	2762.05202	-0.2973896	0.00093949
Rgma	684.917266	-0.2994165	0.0046996
Shisa7	1081.41525	-0.3007019	0.0046996
Slc6a11	2952.28572	-0.3010921	0.0011208

Tmem94	825.062868	-0.301188	0.00559951
Cbarp	2864.984	-0.3012967	0.00096569
Foxg1	724.158055	-0.3016019	0.02285662
Polr1a	394.976351	-0.3026821	0.01302628
Sez6	2020.82716	-0.3056765	0.00719293
Cacng4	468.048684	-0.3077436	0.01123544
Adgrb2	3474.74825	-0.3081928	0.0088412
Ccdc92b	376.373071	-0.3084327	0.03406097
Phf2	730.816231	-0.3093277	0.00369641
Nptx2	234.859152	-0.312525	0.04981141
Kcnj4	604.425963	-0.3160803	0.04267558
Nrp2	384.109487	-0.3194527	0.01090116
Ssbp4	734.429567	-0.3216692	0.00202836
Atn1	1911.86384	-0.3230634	0.00128245
Zfp319	282.248826	-0.3247405	0.02236469
Rgs9	548.842026	-0.3280885	0.02782402
Adcy5	3193.8425	-0.3323958	0.03160297
Grip2	408.08663	-0.3340322	0.00859357
Tox2	338.58464	-0.3362557	0.02732814
Limk1	1027.25099	-0.3372732	0.00088785
Rasal1	632.856454	-0.3374163	0.00924179
Sema5b	206.905574	-0.3409799	0.0364276
Cpne5	668.44757	-0.3485985	0.0168916
Cactin	272.15381	-0.349578	0.02236469
Cacng8	499.239142	-0.3544479	0.0092212
Slit1	787.597902	-0.3568099	0.00136166
Dscaml1	549.599862	-0.3731594	0.00131444
Cacna1h	816.660673	-0.4001402	0.00037146
Hcn4	127.625755	-0.4075674	0.04860764
Baiap3	714.167006	-0.410072	0.01463513
Pcdhgc4	320.631477	-0.4202589	0.02990791
Zfp628	126.497466	-0.4520289	0.04542842
Fzd2	120.735636	-0.4891012	0.02790403
